# Evolutionary Multitasking-Based Multiobjective Optimization Algorithm for Channel Selection in Hybrid Brain Computer Interfacing Systems

**DOI:** 10.3389/fnins.2021.749232

**Published:** 2021-10-05

**Authors:** Tianyu Liu, Zhixiong Xu, Lei Cao, Guowei Tan

**Affiliations:** ^1^School of Information Engineering, Shanghai Maritime University, Shanghai, China; ^2^Department of Neurosurgery, Xiamen Key Laboratory of Brain Center, The First Affiliated Hospital of Xiamen University, Xiamen, China

**Keywords:** brain-computer interfaces, channel selection, evolutionary multitasking, multiobjective optimization, two-stage framework

## Abstract

Hybrid-modality brain-computer Interfaces (BCIs), which combine motor imagery (MI) bio-signals and steady-state visual evoked potentials (SSVEPs), has attracted wide attention in the research field of neural engineering. The number of channels should be as small as possible for real-life applications. However, most of recent works about channel selection only focus on either the performance of classification task or the effectiveness of device control. Few works conduct channel selection for MI and SSVEP classification tasks simultaneously. In this paper, a multitasking-based multiobjective evolutionary algorithm (EMMOA) was proposed to select appropriate channels for these two classification tasks at the same time. Moreover, a two-stage framework was introduced to balance the number of selected channels and the classification accuracy in the proposed algorithm. The experimental results verified the feasibility of multiobjective optimization methodology for channel selection of hybrid BCI tasks.

## 1. Introduction

Brain-computer interface (BCI) develops a communication means between brains and external devices (Alcaide-Aguirre and Huggins, 2014). This technology is used for developing assistive devices for helping paralyzed patients or control gaming. Due to its convenience, electroencephalography (EEG) is widely adopted in non-invasive BCIs for multi-channel signal acquisition (Kevric and Subasi, 2017). In general, more channels would provide more information that can improve the performance of BCI classification. However, channel selection is essential to filter redundant information and simplify experimental manipulation. For example, MI tasks may only need a number of channels as few as 3–5 without lowering the classification accuracy according to temporal-spatio analysis (Pfurtscheller and da Silva, 1999). Furthermore, using more channels is inconvenience for clinical operation and signal processing. Therefore, how to make a compromise between channel number and classification accuracy is a worth-probing question, especially for hybrid BCIs in real-world applications.

In previous works, several effective methods have been proposed for channel selection, such as filter-based methods (Yang et al., 2016), wrapper-based methods (Qiu et al., 2016), correlation-based methods (Jin et al., 2019), machine learning methods (Su et al., 2019), Heuristic searching methods (Sun et al., 2021), and so on. As mentioned before, channel selection needs to make a compromise between two objectives: the number of selected channels and classification accuracy. This perfectly coincides with the goal of multiobjective evolutionary algorithms (MOEAs), which is to make a compromise among multiple optimization objectives. In recent years, multiobjective evolutionary algorithms (MOEAs) have demonstrated their effectiveness in channel selection. For example, the classic Non-dominated Sorting Genetic Algorithm-II (NSGA-II), Multiobjective Evolutionary Algorithm Based on Decomposition (MOEA/D), and Multiobjective Particle Swarm Optimization (MOPSO) have been successfully applied for channel selection in the task of single-modality -based BCIs (Hasan et al., 2010; Moubayed et al., 2010; Kee et al., 2015). Current studies show that hybrid BCI, which combines motor imagery (MI) signals and steady-state visual evoked potentials (SSVEPs), can achieve certain goals more efficiently than single-modality -based BCI systems (Long et al., 2012; Ko et al., 2014). However, relevant works only focus on either the MI classification task (Jin et al., 2020; Sun et al., 2021) or the SSVEP classification task (Zhang et al., 2017; Ravi et al., 2019). Few works conduct channel selection for hybrid BCI tasks.

In this paper, a multitasking-based multiobjective evolutionary algorithm (EMMOA) is proposed to perform channel selection for MI and SSVEP tasks at the same time. In EMMOA, the problems of channel selection for both MI and SSVEP tasks can be regarded as multi-objective optimization problems (MOPs). In order to make a balance between classification accuracy and channel number, the problem of channel selection for MI task can be formulated as a two-objective optimization problem, which contains two objectives: the classification accuracy for MI task and the number of selected channels. Evolutionary multitasking mechanism, which is inspired by bio-cultural models of multifactorial inheritance (Gupta et al., 2015, 2016), provides the investigation of solving multiple tasks via a single population concurrently. In the evolutionary multitasking mechanism, different tasks will experience information transfer during the evolution process since they use the same population. Therefore, if multiple tasks are related then the searching process of solving one task may offer help in solving the other tasks (Gong et al., 2019; Bai et al., 2021). As the evolutionary multitasking mechanism is proved to be an efficient way to optimize multiple tasks, it has been introduced in EMMOA to optimize MI and SSVEP tasks simultaneously in this paper. Furthermore, EMMOA adopts a two-stage framework to improve searching efficiency. The first stage is based on an evolutionary multitasking mechanism and aims to obtain the Pareto-optimal solutions (PS) for MI and SSVEP tasks by one single population. The second stage is local searching. The second stage constructs a three-objective optimization problem, which used classification accuracy for MI task, classification accuracy for SSVEP task, and the number of selected channels as the optimization objectives. The constructed three-objective problem is optimized according to decision variable analysis based on the results obtained by the first stage.

The rest of this paper is organized as follows: Section 2 elaborates the data set, pre-processing and optimization methods used in this paper. Section 3 presents experimental and result analysis, followed by further discussion of this paper.

## 2. Materials and Methods

### 2.1. Subjects and Data Acquisition

**Figure 1:**
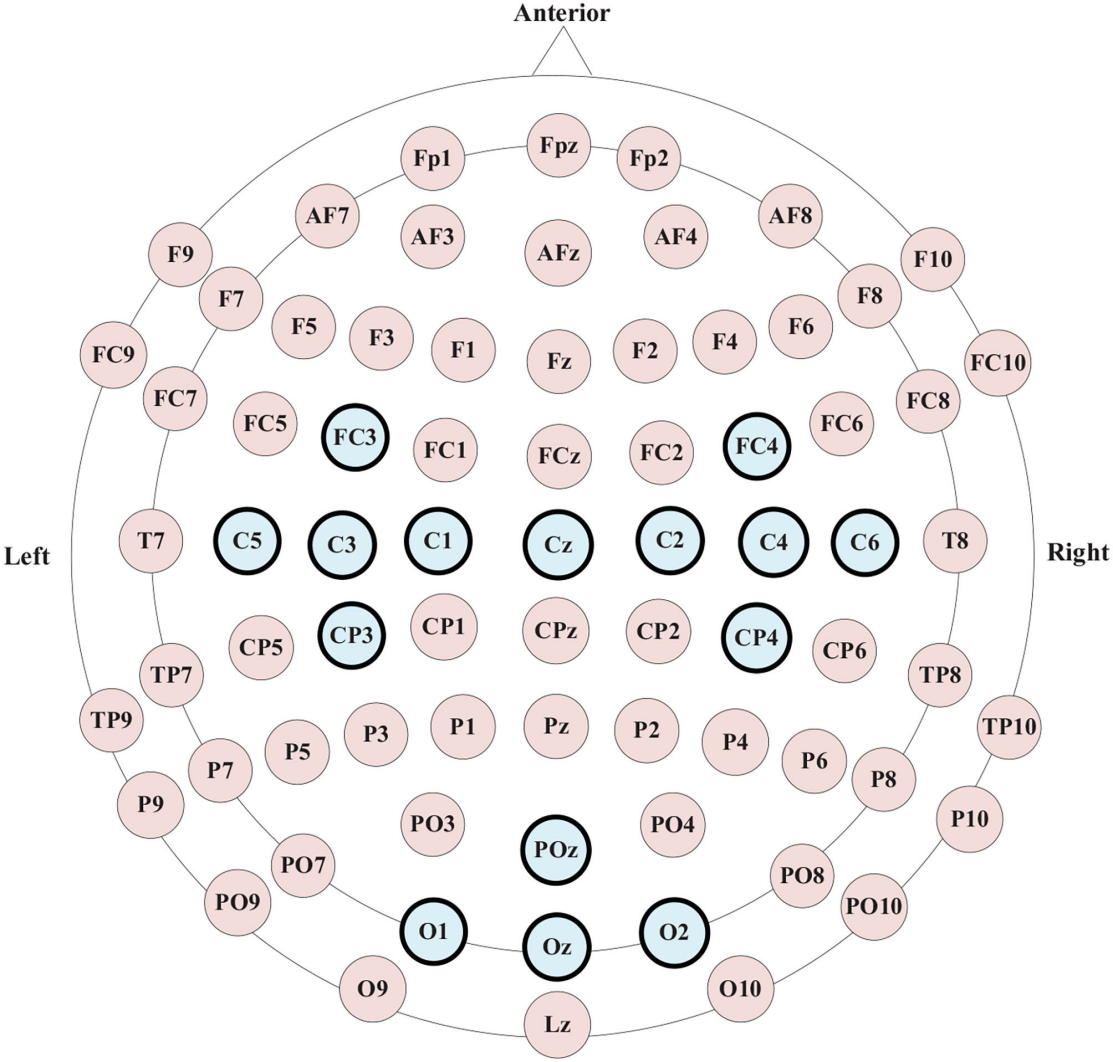
Names and distribution of EEG electrodes.

As shown in Figure 1, fifteen electrodes (i.e., “FC3,” “FC4,” “C5,” “C3,” “C1,” “Cz,” “C2,” “C4,” “C6,” “CP3,” “CP4,” “POz,” “O1,” “Oz,” and “O2”) were used to acquire EEG signals by using a high-performance bio-signal amplifier. These electrodes were placed at the frontal, central, parietal and occipital regions. And the impedances were kept below 5 kΩ. EEG data was sampled at a frequency of 256 Hz and band pass filtered at 0.1–30 Hz.

In this experiment, 7 healthy volunteers, aged from 21 to 30, were selected for data acquisition. Subjects had no prior experience with the operation of hybrid BCI. They all gave informed consent approved by the Ethics Committee.

### 2.2. Feature Selection and Classification

In our work, MI tasks including left- and right-hand imagery are performed for pattern recognition. EEG signals are band pass filtered from 5 to 30 Hz firstly. Then the features are extracted by a well-known common spatial pattern (CSP) algorithm. The CSP method is useful for discriminating two populations of EEG dataset. The detailed methodology can be reviewed in Muller-Gerking et al. (2004). And a radial basis function kernel support vector machine (RBF-SVM) classifier is used for training these feature vectors.

From Lin et al. (2006), we utilize the canonical correlation analysis (CCA) method for SSVEP detection. CCA reflected the correlation relationship between EEG response signals and classical Fourier series at the stimulus frequency and its harmonics. It is widely used for spectral analysis in the biosignal process.

### 2.3. Multi-Objective Channel Selection Problem Formulation

Suppose the number of total channels is *K*, then a solution for the channel selection problem can be defined as a *K*-dimensional vector *x*, as shown in Equation (1). *x*_*i*_(1 ≤ *i* ≤ *K*) is called a decision variable. *x*_*i*_ = 0 means the corresponding channel is not selected. Otherwise, the corresponding channel is selected.


(1) $$\begin{array}{r} x=\left[x_{1},x_{2},...,x_{K}\right],x_{i}\in \left\{0,1\right\} \end{array}$$

The purpose of channel selection problems is to select a smaller set of channels with as little sacrifice as possible in classification accuracy. For the MI classification task, the first objective function is the MI classification accuracy rate (*MAR*). The second objective function is defined as *NC* = *K* − *C*, where *C* is the number of selected channels (i.e., the number of elements whose value is 1 in solution *x*). Therefore, the ideal situation for an optimization algorithm is obtaining an optimal solution x^*^ that has the maximum values for both *MAR* and *NC*. However, it is impossible to get the above-mentioned x^*^, since the confliction between *MAR* and *NC*. Thus, evolutionary optimization algorithms will obtain a Pareto Set (PS), which contains a set of Pareto-optimal solutions (non-dominated solutions) (Wang et al., 2018). One solution *x* is called a Pareto-optimal solution, if and only if there not exist any other solutions that are better than *x* for all objective functions. The Pareto optimal solutions are incomparable, since solution *x* may be better than solution *y* for one objective function but worse than *y* for the other one.

Similarly, the two objectives for SSVEP classification task are the SSVEP classification accuracy rate (*SAR*) and *NC*. In the proposed algorithm, the second stage aims to improve the optimization performance for both MI and SSVEP tasks by local searching, therefore a three-objective optimization problem, which uses *MAR*, *SAR*, and *NC* as the objective functions, is adopted in this stage.

### 2.4. Evolutionary Multitasking-Based Multiobjective Optimization Algorithm (EMMOA)

**Figure 2:**
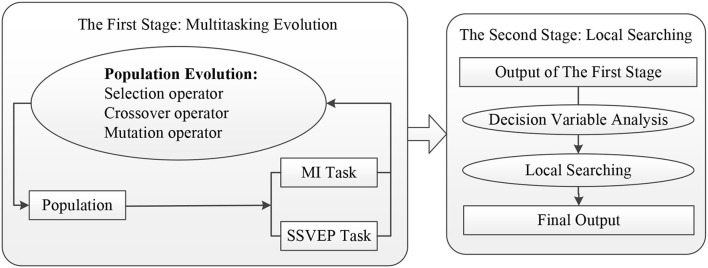
Framework of EMMOA.

The framework of EMMOA is given in Figure 2. As shown in Figure 2, EMMOA adopts a two-stage framework. In EMMOA, the first stage is designed according to evolutionary multitasking mechanism and uses one single population to optimize two tasks (MI task and SSVEP task) simultaneously. In this case, the two tasks will experience information transfer during the evolution process since they use the same population. The information transfer can share underlying similarities between the two tasks thereby facilitating improved optimization performance for both MI and SSVEP tasks. Based on the output of the first stage, the second stage aims to obtain the final output of the algorithm. With the help of decision variables analysis, the local searching strategy in the second stage makes a better compromise among MI classification accuracy, SSVEP classification accuracy, and the number of selected channels. More specifically, in the second stage, a decision variable analysis operator will be carried out on the Pareto-optimal sets obtained by the first stage and aims to divide the decision variables into different groups. The local searching operator will determine the searching directions of individuals according to the type of each decision variable and help the algorithm search more efficiently. The detailed description of the two stages is given below.

In the first stage, suppose the population size is *N* and the total number of channels is *K*, then the individual population can be initialized by generating *N* individuals (solutions) and each individual contains *K* elements. In this case, the individual population can be initialized as a *N* × *K* matrix. Each element in the individual population is uniformly generated from [0, 1]. If the value of an element is larger than 0.5, then the element will take the value of 1. Otherwise, the element will be set to 0. By associating each individual with a task label by the task assignment operator. In the initialization step, the task assignment operator is carried out by giving each individual the label, which is generated from {1, 2} randomly. In the evolution process, the task labels of individuals are inherited from their parents. Each individual will optimize the task that is specified by its task label. For a task label, 1 and 2 represent the corresponding individual is assigned to optimize MI task and SSVEP task, respectively. The individual population is updated by using the population evolution step, which includes the tournament selection operator (Zhang et al., 2016), partial-mapped crossover operator (Ismkhan and Zamanifar, 2015), and flip-bit mutation operator (Chicano et al., 2015). The tournament selection operator, partial-mapped crossover operator, and simple mutation operator are classic operators, which are widely adopted in evolutionary algorithms to generate offspring populations (lines 1–21 in Algorithm 1). The task labels of offspring individuals are inherited from their parents in the evolution process (line 7 and line 19 in Algorithm 1). After updating the individual population (lines 22–23 in Algorithm 1), the Pareto-optimal sets for MI and SSVEP tasks will be updated accordingly (line 24 in Algorithm 1). Therefore, the first stage will output two Pareto-optimal sets (namely PS_MI and PS_SSVEP) for MI and SSVEP tasks separately. PS_MI contains a set of non-dominated solutions which aim to optimize *MAR* and *NC*, while PS_SSVEP contains a set of non-dominated solutions which aim to optimize *SAR* and *NC*. In this paper, the maximum size of both PS_MI and PS_SSVEP is set to 100. Take PS_MI for an example, if the size of PS_MI exceeds 100, then the non-dominated solutions in PS_MI will be decreased according to Crowding Distance (Deb et al., 2002). Crowding Distance, which is proposed to estimate the density of solutions, is widely adopted to maintain population diversity in EAs. The solution with a larger Crowding Distance value can be considered to make great contributions for maintaining population diversity. Therefore, if the number of the solutions in PS_MI exceeds 100, then the members with larger Crowding Distance values will be remain.

**Algorithm 1:** First stage of EMMOA.

**Input:**
POP (the individual population)
PS_MI and PS_SSVEP (the original Pareto-optimal sets for MI and SSVEP tasks)
**Output:**
PS_MI and PS_SSVEP (the updated Pareto-optimal sets for MI and SSVEP tasks)
1: Generate parent population (parent_POP) by tournament selection operator from POP;
2: *i* = 1, offspring_POP = ∅;
3: **while** *i* < size(parent_POP) **do**
4: Select parent_POP(*i*) and parent_POP(*i* + 1) as parent1 and parent2, respectively;
5: **if** task_label(parent1)=task_label(parent2) **then**
6: Generate two offspring individuals (offspring1 and offpring2) according to partial-mapped crossover operator;
7: task_label(offspring1)=task_label (parent1), task_label(offspring2) = task_label (parent2);
8: **else**
9: Obtain *r*_1_ which is randomly generated from [0, 1];
10: **if** *r*_1_ is smaller than the predefined crossover probability **then**
11: Generate two offspring individuals (offspring1 and offpring2) according to partial-mapped crossover operator;
12: **else**
13: Obtain *r*_2_ which is randomly generated from [0, 1];
14: **if** *r*_2_ is smaller than the predefined mutation probability **then**
15: Generate two offspring individuals (offspring1 and offpring2) according to flip-bit mutation operator;
16: **end if**
17: **end if**
18: **end if**
19: task_label(offspring1) = task_label (parent1), task_label(offspring2) = task_label (parent2);
20: offspring_POP = offspring_POP ∪ { offspring1,offspring2 };
21: **end while**
22: temp_POP = POP ∪ offspring_POP;
23: Obtain the next POP by selecting the fittest individuals from temp_POP
24: Update PS_MI as the non-dominated solutions from the original PS_MI and the individuals with task_label = 1 in POP and update PS_SSVEP as the non-dominated solutions from the original PS_SSVEP and the individuals with task_label = 2 in POP.

The second stage is implemented based on the output of the first stage, i.e., the current Pareto-optimal sets for MI and SSVEP tasks (PS_MI and PS_SSVEP). The purpose of the second stage is to obtain a set of Pareto-optimal solutions that make a compromise among MI classification accuracy, SSVEP classification accuracy, and the number of selected channels. Therefore, in this stage, the optimization problem becomes a maximum three-objective optimization with *MAR*, *SAR*, and *NC* as its objective functions. In this case, the Pareto-optimal set outputted by the whole algorithm consists of a set of non-dominated solutions which aim to make a compromise among *MAR*, *SAR*, and *NC*. To improve the searching efficiency, a local searching operator, which is based on a decision variable analysis strategy, is introduced in this stage.

The main idea of the proposed decision variable analysis operator is to divide the decision variables into three groups: add_group, delete_group, and invalid_group. The division of the first 11 and the last 4 decision variables are based on PS_SSVEP and PS_MI, respectively. Take the jth (1 ≤ *j* ≤ 11) decision variable for an example. At first, initialize *flag* to 0. For a non-dominated solution (*x*_*i*_) in PS_SSVEP, if the classification accuracy is raised with *x*_*i*_(*j*) = 0, then one can consider the performance of *x*_*i*_ will be improved without selecting the jth channel. In this case, *flag* = *flag* − 1. If *x*_*i*_(*j*) = 1 gets a better classification accuracy, then *flag* = *flag* + 1. If the classification accuracy is not affected by *x*_*i*_(*j*), then the value of *flag* will remain unchanged. All the members in PS_SSVEP will be executed to get the final value of *flag*.The jth decision variable will be divided into different groups according to the value of *flag*. The detailed procedure of the proposed decision variable analysis operator is given in Algorithm 2.

**Algorithm 2:** Detailed procedure of division variable analysis operator.

**Input:**
PS_MI and PS_SSVEP
n1(number of the solutions in PS_MI), n2(number of the solutions in PS_SSVEP)
**Output:**
add_group, delete_group, and invalid_group
1: add_group=∅, delete_group = ∅, invalid_group = ∅;
2: **for** *j* = 1 TO 11 **do**
3: *flag* = 0;
4: **for** *i* = 1 TO n1 **do**
5: *x* is the *i*^*th*^ solution in PS_MI, *x*1 = *x*;
6: *x*_*MAR* is the MI classification accuracy of the *i*^*th*^ solution in PS_MI;
7: **if** *x*1(*j*) = 0 **then**
8: *x*1(*j*) = 1 and *x*1_*MAR* is the MI classification accuracy of *x*1;
9: **if** *x*_*MAR*< *x*1_*MAR* **then**
10: *flag* = *flag* + 1 ;
11: **else if** *x*_*MAR* > *x*1_*MAR* **then**
12: *flag* = *flag* − 1 ;
13: **end if**
14: **else**
15: *x*1(*j*) = 0 and *X*1_*MAR* is the MI classification accuracy of *x*1;
16: **if** *x*_*MAR*< *x*1_*MAR* **then**
17: *flag* = *flag* − 1 ;
18: **else if** *x*_*MAR* > *x*1_*MAR* **then**
19: *flag* = *flag* + 1 ;
20: **end if**
21: **end if**
22: **end for**
23: **if** *flag*>0 **then**
24: add_group = add_group ∪ {*i*};
25: **else if** flag < 0 **then**
26: delete_group = delete_group ∪ {*i*};
27: **else**
28: invalid_group = invalid_group ∪ {*i*};
29: **end if**
30: **end for**

After decision variable analysis, the local searching operator is implemented on PS_MI and PS_SSVEP. The detailed procedure of the proposed local searching operator is shown in Algorithm 3. For the Pareto-optimal solutions in PS_MI, the searching directions are obtained according to the types of the last four decision variables (lines 2–11 in Algorithm 3). For the Pareto-optimal solutions in PS_SSVEP, the searching directions are obtained according to the types of the first eleven decision variables (lines 12–21 in Algorithm 3). The current Pareto-optimal set of EMMOA will be acquired by selecting the non-dominated solutions in the original Pareto-optimal set and newPOP obtained by the local searching operator.

**Algorithm 3:** Detailed procedure of local searching operator.

**Input:**
add_group, delete_group, and invalid_group
n1 (number of the solutions in PS_MI), n2 (number of the solutions in PS_SSVEP)
**Output:**
newPOP
1: newPOP=∅;
2: **for** *i* = 1 TO n1 **do**
3: *x* is the *i*^*th*^ solution in PS_MI, *x*1 = *x*;
4: *r* is a random integer from [12, 15];
5: **if** *r* ∈ add_group and *x*(*i*) = 0 **then**
6: *x*1(*i*) = 1;
7: **else if** *r* ∈ delete_group and *x*(*i*) = 1 **then**
8: *x*1(*i*) = 0;
9: **end if**
10: newPOP = newPOP ∪*x*1;
11: **end for**
12: **for** *i* = 1 TO n2 **do**
13: *x* is the *i*^*th*^ solution in PS_SSVEP, *x*1 = *x*;
14: *r* is a random integer from [1, 11];
15: **if** *r* ∈ add_group and *x*(*i*) = 0 **then**
16: *x*1(*i*) = 1;
17: **else if** *r* ∈ delete_group and *x*(*i*) = 1 **then**
18: *x*1(*i*) = 0;
19: **end if**
20: newPOP = newPOP ∪*x*1;
21: **end for**

## 3. Experiment and Result Analysis

### 3.1. Experimental Setup

This section contained two experiments. The first one aimed to demonstrate whether the proposed channel selection algorithm (EMMOA) could obtain a smaller set of channels with as little sacrifice as possible in MI and SSVEP classification accuracy. The second one aimed to evaluate the effectiveness of EMMOA by comparing with three widely used multiobjective optimization algorithms, including NSGA-II (Deb et al., 2002), MOEA/D (Zhang and Li, 2007), and MOPSO (Coello et al., 2004). The number of total function evaluations was 10000 in a single run for all 4 algorithms. The detailed parameter settings of algorithms were given in Table 1.

**Table 1:** Parameter settings of algorithms.

**Algorithm**	**Parameter settings**
EMMOA	Population size: 100; crossover parameter: 0.8; mutation probability: 0.2
NSGA-II	Population size: 100
MOEA/D	Population size: 105
MOPSO	Population size: 100

### 3.2. Results and Analysis

**Figure 3:**
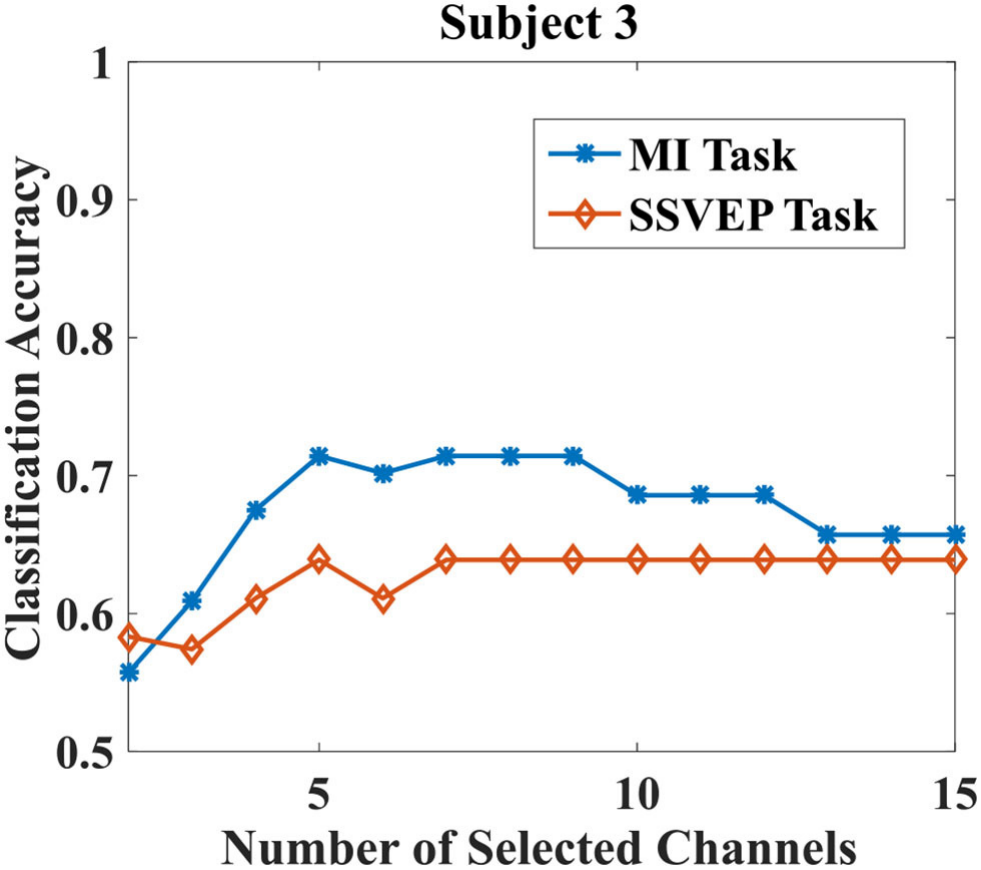
Classification accuracies for MI and SSVEP tasks with different numbers of selected channels.

**Figure 4:**
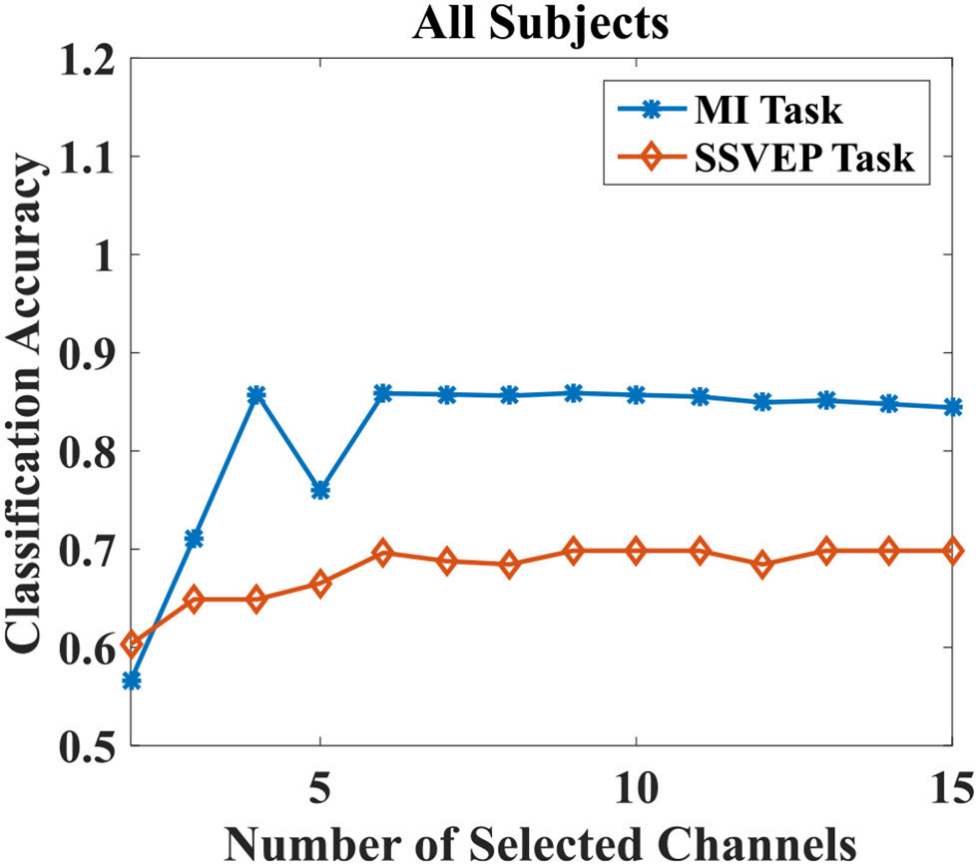
Average classification accuracies for MI and SSVEP tasks with different numbers of selected channels for all subjects.

The purpose of the proposed EMMOA is to find the optimum number of channels that would result in a promising performance for both MI and SSVEP tasks. Take subject 3 as an example, Figure 3 illustrated the classification accuracies for MI and SSVEP tasks with different numbers of selected channels. It can be observed from Figure 3, the classification accuracy for MI task tends to become higher at first and then become lower as the number of selected channels increases. The classification accuracy for SSVEP task tends to be stable when the number of selected channels. Figure 4 illustrated the average classification accuracies for all 7 subjects with different numbers of selected channels. Figure 4 demonstrated that the larger number of selected channels cannot lead to better performance for MI and SSVEP tasks. This may indicate the necessity of selecting a promising subset of channels.

Table 2 demonstrated the classification accuracy for MI and SSVEP tasks by using all channels and the reduced set of EEG channels selected by the proposed EMMOA. As Table 2 shows, EMMOA can find a smaller set of channels without depredating the classification performance for all 7 subjects. For example, the classification accuracies for MI and SSVEP tasks using 5 channels selected by EMMOA are better than those using all channels. For subject 6, the classification performance using all channels is same to that of using 5 channels. However, a smaller number of channels is certainly more convenient for real-life applications. Table 3 gives the average classification accuracies for all subjects by using all channels and the Pareto-optimal set obtained by EMMOA in a single run. It can be observed from Table 3, the classification performance by using 6, 7, and 8 channels selected by EMMOA is better than using all channels. Therefore, the experimental results demonstrated the effectiveness of using the proposed channel selection algorithm.

**Table 2:** Classification accuracies for each subject by using all channels and the selected channels obtained by EMMOA.

**Subject**	**All Channels**		**Using EMMOA**		
	**MAR (%)**	**SAR (%)**	**MAR (%)**	**SAR (%)**	**Number of selected channels**
Subject-1	1	0.6111	1	0.6667	4
Subject-2	0.5824	0.5	0.6574	0.5833	5
Subject-3	0.6571	0.6388	0.7143	0.6389	5
Subject-4	1	0.8611	1	0.9444	4
Subject-5	0.67	0.5833	0.75	0.5833	4
Subject-6	1	0.7222	1	0.7222	5
Subject-7	1	0.9722	1	1	4

**Table 3:** Average classification accuracies for all subjects by using all channels and the pareto-optimal set obtained by EMMOA for all subjects.

**All channels**		**PS by EMMOA**		
**MAR (%)**	**SAR (%)**	**MAR (%)**	**SAR (%)**	**Number of selected channels**
0.8442	0.6984	0.5661	0.6032	2
		0.5661	0.6944	3
		0.8490	0.6032	3
		0.8561	0.5893	3
		0.8561	0.6944	4
		0.8597	0.6012	4
		0.5661	0.6984	5
		0.8597	0.6944	5
		0.8561	0.6984	6
		0.8581	0.6984	7
		0.8611	0.6468	7
		0.8611	0.6984	8

For further comparison of the proposed EMMOA with other classical multi-objective evolutionary algorithms, three algorithms, including NSGA-II, MOEA/D and MOPSO, are adopted in this section. All the comparative algorithms have been running 30 times to get the statistical results, which are shown in Table 4. In Table 4, the numbers in brackets indicate the rank of the corresponding algorithm in terms of average MI accuracy, average SSVEP accuracy, and average number of selected channels, respectively. A smaller rank value of an algorithm means a better performance achieved by the algorithm. In the last column of Table 4, EMMOA obtained the smallest (best) average rank. In other words, the proposed channel selection algorithm achieved the best performance in terms of average rank. Table 5 gives the maximum average classification accuracies for MI and SSVEP tasks in the PS obtained by all the 4 algorithms with different numbers of selected channels. The numbers in boldface give the best accuracy rates in different cases. It can be observed from Table 5, EMMOA obtained the best classification accuracies for all 3 cases.

**Table 4:** Average results obtained by all algorithms.

**Algorithm**	**Average MAR (%)**	**Average SAR (%)**	**Average number of selected channels**	**Average rank**
NSGA-II	0.7606 (4)	0.6601 (2)	4.48 (1)	2.33
MOEA/D	0.8480 (1)	0.6586 (2)	6.67 (4)	2.67
MOPSO	0.7684 (3)	0.6553 (4)	4.83 (3)	3.33
EMMOA	0.7969 (2)	0.6653 (1)	4.57 (2)	1.67

**Table 5:** Maximum results obtained by all algorithms.

**Algorithm**	**Maximum MAR (%)**	**Maximum SAR (%)**	**Number of selected channels**
NSGA-II	**0.8561**	0.6032	3
MOEA/D	0.8362	0.6032	
MOPSO	0.5661	**0.6944**	
EMMOA	**0.8561**	**0.6944**	
NSGA-II	0.8597	0.6944	5
MOEA/D	0.8526	0.6548	
MOPSO	0.8597	**0.6984**	
EMMOA	**0.8611**	**0.6984**	
NSGA-II	**0.8611**	**0.6984**	7
MOEA/D	0.8397	**0.6984**	
MOPSO	0.8597	**0.6984**	
EMMOA	**0.8611**	**0.6984**	

The results in Tables 4, 5 demonstrated the effectiveness of the proposed algorithm. This may be because the multitasking mechanism adopted in the first stage of EMMOA help the algorithm find more promising PS for each task efficiently. On the other hand, with the help of decision variables analysis, the local searching strategy in the second stage makes a better compromise among MI classification accuracy, SSVEP classification accuracy, and the number of selected channels.

**Figure 5:**
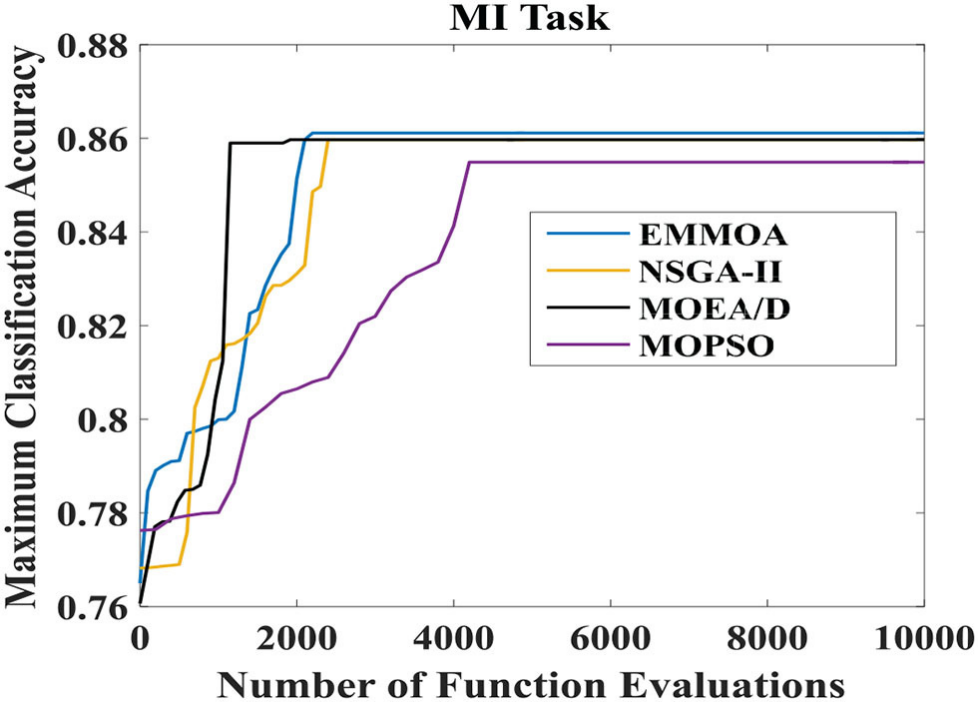
Convergence of four algorithms for MI task.

**Figure 6:**
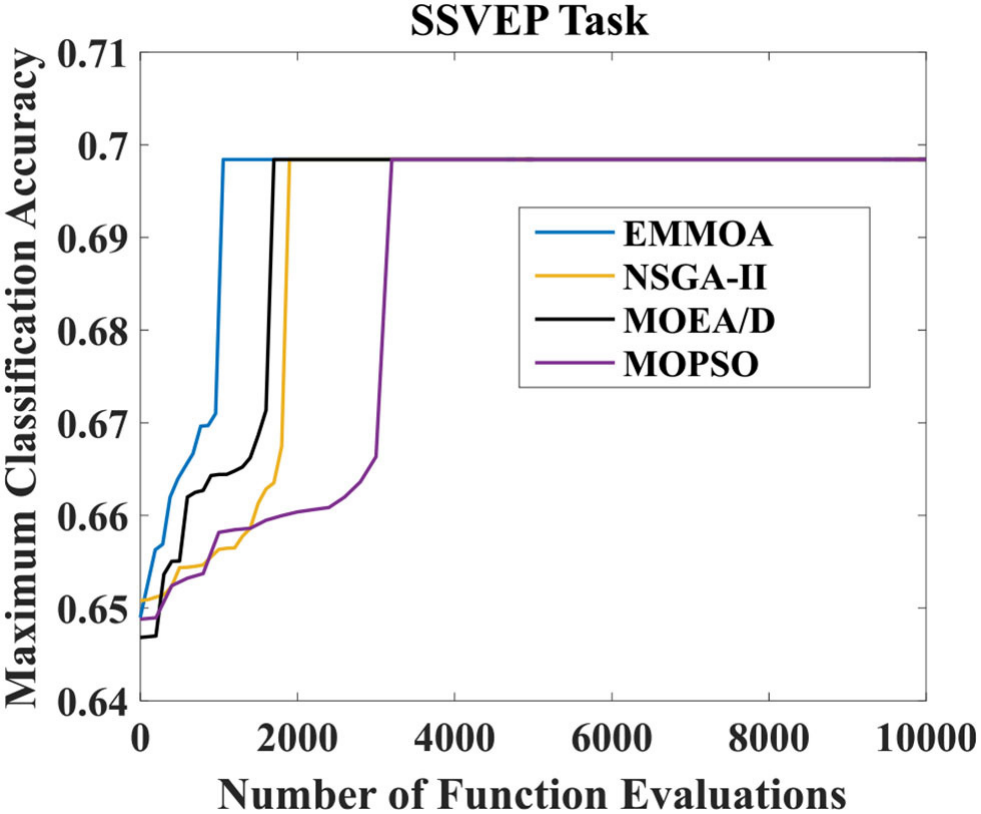
Convergence of four algorithms for SSVEP task.

Figures 5, 6 illustrated the convergence of four algorithms for MI and SSVEP tasks, respectively. In Figures 5, 6, the x-axis represented the number of function evaluations and the y-axis displayed the maximum classification accuracy averaged over all the 7 subjects. As Figure 5 showed, the proposed EMMOA obtained the second-best and the best convergence speed for MI and SSVEP tasks among four algorithms, respectively. This is because the decision variable analysis strategy adopted in EMMOA helped the algorithm searching more efficiently.

## 4. Discussion

It can be observed from Figure 4, the best classification accuracy can be achieved when the number of selected channels is not very large. This observation can be validated by the results demonstrated in Table 3. The classification accuracies for both MI and SSVEP tasks are higher than 0.65 when the number of selected channels is larger than 3. As Figure 4 showed, the improvement in classification accuracy is not obvious by adopting 4 channels when compared with using more than 4 channels. Especially for MI task, the classification accuracy by using 4 channels is even better than using all 15 channels. The phenomenon is in agreement with the conclusion in Moubayed et al. (2010), which showed that some channels may bring artifacts and then lead to a degeneration in classification accuracy.

In the proposed algorithm, the decision variable analysis operator played a vital role in the second stage of EMMOA, since it provided searching directions for the next local searching operator. Table 6 showed the results outputted by the decision variable analysis operator in the middle and last stages of the optimization process by using EMMOA. add_group, delete_group and invalid_group contains a set of channels that are considered to be profitable, disadvantageous, and invalid for the corresponding classification task. As Table 6 shows, most results obtained by the decision variable analysis operator in the middle stage overlap with those obtained in the last stage. However, the results in different stages are not exactly the same. This phenomenon may indicate the uncertainty of the decision variable analysis operator to some extent. As described in section 2.4, the performance of the second stage of EMMOA depends on the results of the proposed decision variable analysis operator. Therefore, the second stage may not obtain a promising output if the decision variable analysis operator cannot get an appropriate division of the decision variables, especially in the early stage of the evolution process. How to improve the stability of the proposed decision variable analysis operator will be the next work of this paper.

**Table 6:** Results outputted by decision variable analysis operator.

**First 11 channels (for MI Task)**		**Last 4 channels (for SSVEP Task)**	
**Middle Stage of EMMOA**			
add_group	{2, 3, 5, 8, 9}	add_group	{12, 13, 14, 15}
delete_group	{1, 7, 10, 11}	delete_group	{}
invalid_group	{6}	delete_group	{}
**Last stage of EMMOA**			
add_group	{2, 3, 4, 7, 8, 9}	add_group	{12, 13, 14, 15}
delete_group	{1, 5, 6, 10, 11}	delete_group	{}
invalid_group	{}	delete_group	{}

**Figure 7:**
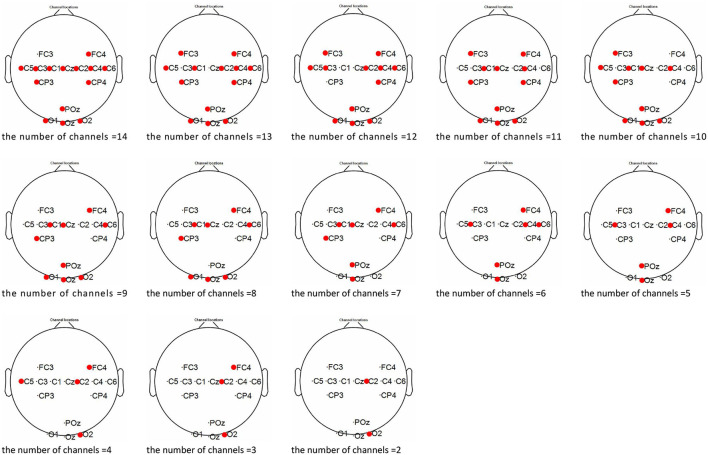
The distribution diagrams in conditions of different numbers of selected channels.

As shown in Figure 7, the distribution diagrams were illustrated in conditions of different numbers of selected channels. Evidently, the electrodes were first eliminated from motor cortex when the number was sufficient for MI and SSVEP recognition. And the quantity of channels were close between central area and occipital area along with the decline of total number. Meanwhile, the priority of selecting was unclear in these two regions because of the alternate quantitative superiority. However, the feature information needed to be reserved those extracted from left- and right- motor cortex for spatial filtering. Thus, the limitation of channel number in the occipital area might be firstly considered as the kernel factor of EMMOA.

## 5. Conclusion

In our study, a multitasking-based multiobjective evolutionary algorithm (EMMOA) was proposed to select appropriate channels for these two classification tasks at the same time. Moreover, a two-stage framework was introduced to balance the number of selected channels and the classification accuracy in the proposed algorithm. The experimental results verified the feasibility of multiobjective optimization methodology for channel selection of hybrid BCI tasks.

## Data Availability Statement

The raw data supporting the conclusions of this article will be made available by the authors, without undue reservation.

## Ethics Statement

The studies involving human participants were reviewed and approved by Ethics Committee of Tongji University. The patients/participants provided their written informed consent to participate in this study.

## Author Contributions

TL designed the methodology of data process and performed the data analysis. TL and ZX organized the data and wrote the manuscript. LC and GT reviewed and edited the manuscript. All authors read and approved the submitted manuscript.

## Funding

This work was supported by the National Natural Science Foundation of China (grant nos. 61806122 and 62102242).

## Conflict of Interest

The authors declare that the research was conducted in the absence of any commercial or financial relationships that could be construed as a potential conflict of interest.

## Publisher's Note

All claims expressed in this article are solely those of the authors and do not necessarily represent those of their affiliated organizations, or those of the publisher, the editors and the reviewers. Any product that may be evaluated in this article, or claim that may be made by its manufacturer, is not guaranteed or endorsed by the publisher.

## References

[B1] Alcaide-Aguirre R. E. Huggins J. E. (2014). Novel hold-release functionality in a p300 brain-computer interface. J. Neural Eng. 11:066010. 10.1088/1741-2560/11/6/066010 25380071 PMC4843815

[B2] Bai L. Lin W. Gupta A. Song Y. S. (2021). From multitask gradient descent to gradient-free 15 evolutionary multitasking: a proof of faster convergence. IEEE Trans. Cybern. 10.1109/TCYB.2021.3052509. [Epub ahead of print]. 33705329

[B3] Chicano F. Sutton A. M. Whitley L. D. Alba E. (2015). Fitness probability distribution of bit-flip mutation. Evol. Comput. 23, 217–248. 10.1162/EVCO_a_00130 24885680

[B4] Coello C. A. C. Pulido G. T. Lechuga M. S. (2004). Handling multiple objectives with particle swarm optimization. IEEE Trans. Evol. Comput. 8, 256–279. 10.1109/TEVC.2004.826067

[B5] Deb K. Pratap A. Agarwal S. Meyarivan T. (2002). A fast and elitist multiobjective genetic algorithm: Nsga-ii. IEEE Trans. Evolut. Comput. 6, 182–197. 10.1109/4235.996017

[B6] Gong M. Tang Z. Li H. Zhang J. (2019). Evolutionary multitasking with dynamic resource allocating strategy. IEEE Trans. Evolut. Comput. 23, 858–869. 10.1109/TEVC.2019.2893614

[B7] Gupta A. Ong Y. S. Feng L. (2015). Multifactorial evolution: toward evolutionary multitasking. IEEE Trans. Evolut. Comput. 20, 343–357. 10.1109/TEVC.2015.2458037

[B8] Gupta A. Ong Y. S. Feng L. Tan K. C. (2016). Multiobjective multifactorial optimization in evolutionary multitasking. IEEE Trans. Cybern. 47, 1652–1665. 10.1109/TCYB.2016.2554622 27164616

[B9] Hasan B. A. S. Gan J. Q. Zhang Q. (2010). Multi-objective evolutionary methods for channel selection in brain-computer interfaces: some preliminary experimental results, in IEEE Congress on Evolutionary Computation (Barcelona: IEEE), 1–6.

[B10] Ismkhan H. Zamanifar K. (2015). Study of some recent crossovers effects on speed and accuracy of genetic algorithm, using symmetric travelling salesman problem. arXiv preprint arXiv: 1504.02590.

[B11] Jin J. Liu C. Daly I. Miao Y. Li S. Wang X.. (2020). A hybrid brain computer interface to control the direction and speed of a simulated or real wheelchair. IEEE Trans. Neural Syst. Rehabil. Eng. 28, 2153–2163. 10.1109/TNSRE.2020.3020975 22692936

[B12] Jin J. Miao Y. Y. Daly I. Zuo C. L. Hu D. W. Cichocki A. (2019). Correlation-based channel selection and regularized feature optimization for mi-based bci. Neural Netw. 118, 262–270. 10.1016/j.neunet.2019.07.008 31326660

[B13] Kee C. Y. Ponnambalam S. G. Loo C. K. (2015). Multi-objective genetic algorithm as channel selection method for p300 and motor imagery data set. Neurocomputing 161, 120–131. 10.1016/j.neucom.2015.02.057

[B14] Kevric J. Subasi A. (2017). Comparison of signal decomposition methods in classification of eeg signals for motor-imagery bci system. Biomed. Signal Process. Control 31, 398–406. 10.1016/j.bspc.2016.09.007

[B15] Ko L. W. Lin S. C. Song M. S. Komarov O. (2014). Developing a few-channel hybrid bci system by using motor imagery with ssvep assist, in 2014 International Joint Conference on Neural Networks (IJCNN) (Beijing: IEEE), 4114–4120.

[B16] Lin Z. Zhang C. Wu W. Gao X. (2006). Frequency recognition based on canonical correlation analysis for ssvep-based bcis. IEEE Trans. Biomed. Eng. 53, 261–274. 10.1109/TBME.2006.886577 17152442

[B17] Long J. Li Y. Wang H. Yu T. Pan J. Li F. (2012). A hybrid brain computer interface to control the direction and speed of a simulated or real wheelchair. IEEE Trans. Neural Syst. Rehabil. Eng. 20, 720–729. 10.1109/TNSRE.2012.2197221 22692936

[B18] Moubayed N. A. Hasan B. A. S. Gan J. Q. Petrovski A. (2010). Binary-sdmopso and its application in channel selection for brain-computer interfaces, in 2010 UK Workshop on Computational Intelligence (UKCI) (Colchester: IEEE), 1–6.

[B19] Muller-Gerking J. Pfurtscheller G. Flyvbjerg H. (2004). Designing optimal spatial filters for single-trial eeg classification in a movement task. Clin. Neurophysiol. 110, 787–798. 10.1016/S1388-2457(98)00038-8 10400191

[B20] Pfurtscheller G. da Silva F. H. L. (1999). Handbook of Electroencephalography and Clinical Neurophysiology-Event-Related Desynchronization. Amsterdam: Elsevier.

[B21] Qiu Z. Jin J. Lam H. K. Zhang Y. Cichocki A. (2016). Improved sffs method for channel selection in motor imagery based bci. Neurocomputing 207, 519–527. 10.1016/j.neucom.2016.05.035

[B22] Ravi A. Pearce S. Zhang X. Jiang N. (2019). User-specific channel selection method to improve ssvep bci decoding robustness against variable inter-stimulus distance, in 2019 9th International IEEE/EMBS Conference on Neural Engineering (NER) (San Francisco, CA: IEEE), 283–286.

[B23] Su J. R. Wang J. G. Xie Z. T. Yao Y. Liu J. (2019). A method for eeg contributory channel selection based on deep belief network, in IEEE 8th Data Driven Control and Learning Systems Conference (DDCLS) (Dali: IEEE), 1247–1252.

[B24] Sun H. Jin J. Kong W. Z. Zuo C. L. Li S. R. Wang X. Y. (2021). Novel channel selection method based on position priori weighted permutation entropy and binary gravity search algorithm. Cogn. Neurodyn. 15, 141–156. 10.1007/s11571-020-09608-3 33786085 PMC7947109

[B25] Wang B. C. Li H. X. Zhang Q. Wang Y. (2018). Decomposition-based multiobjective optimization for constrained evolutionary optimization. IEEE Trans. Syst. Man Cybern. Syst. 51, 574–587. 10.1109/TSMC.2018.2876335

[B26] Yang Y. Bloch I. Chevallier S. Wiart J. (2016). Subject-specific channel selection using time information for motor imagery brain-computer interfaces. Cogn. Comput. 8, 505–518. 10.1007/s12559-015-9379-z

[B27] Zhang J. Yan C. Cao L. Gong X. (2017). Optimal channel set selection for ssvep-based bci using spatial temporal correlation, in 2017 13th International Conference on Natural Computation, Fuzzy Systems and Knowledge Discovery (ICNC-FSKD) (Guilin: IEEE), 2038–2042.

[B28] Zhang Q. Li H. (2007). Moea/d: a multiobjective evolutionary algorithm based on decomposition. IEEE Trans. Evolut. Comput. 11, 712–731. 10.1109/TEVC.2007.892759

[B29] Zhang Y. Zhen Y. Lu T. T. (2016). A fuzzy c-means clustering based tournament selection for multiobjective optimization, in 2016 IEEE Congress on Evolutionary Computation (CEC) (Vancouver, BC: IEEE), 2446–2453.

